# Hierarchical bounds on limit cycles in disjunctive and mixed nested canalizing Boolean networks

**DOI:** 10.1007/s12064-026-00498-3

**Published:** 2026-11-09

**Authors:** Belaynneh Yizengaw

**Affiliations:** University of Pretoria, Pretoria, South Africa

**Keywords:** Nested canalizing Boolean networks; Mixed conjunctive–disjunctive networks; Periodic-orbit bounds; Hierarchical admissibility relations; Trap spaces; Exact state-space reduction.

## Abstract

Boolean networks with nested canalizing update rules provide a structured representation of hierarchical regulation, yet structural bounds on periodic dynamics are substantially better developed for homogeneous conjunctive or disjunctive systems than for networks in which these rule types coexist. We develop a synchronous framework for mixed conjunctive–disjunctive nested canalizing Boolean networks and derive certified bounds on their periodic orbits together with exact state-space reductions on invariant trap spaces. The central result constructs a hierarchy of admissibility relations that retains successively deeper canalizing layers and proves that each additional layer can only tighten the corresponding cycle bound, with an exact criterion for strict improvement. On every forward-invariant saturated trap space, the dynamics is conjugate to a reduced Boolean system whose state space decreases exponentially with the number of frozen coordinates. We further give a saturation algorithm and distinguish the polynomial cost of closing a specified seed from the potentially combinatorial problem of enumerating relevant trap spaces. Nonconstant disjunctive and genuinely mixed examples illustrate the theory, while a synthetic five-node example and a scope audit of traceable biological models clarify its present applicability and limitations. The framework thus provides a progressively refinable structural approach to periodic dynamics in mixed canalizing networks while making its computational and biological scope explicit.

## Introduction

Boolean networks provide a finite-state framework for modelling regulatory and logical systems, with applications ranging from discrete dynamical systems to gene-regulatory networks (Kauffman 1969; Drossel 2008). Among structured Boolean rules, canalizing and nested-canalizing functions (NCFs) are particularly important because their hierarchical input–output organization is associated with reduced sensitivity, robustness, and regulatory structure (Jarrah et al. 2007; Li et al. 2013; Kadelka et al. 2017; Dimitrova et al. 2022; Çoban and Kabakçıoğlu 2022; Kadelka et al. 2024a, b). Their structure also offers an opportunity to constrain long-term dynamics without exhaustive exploration of the full Boolean state space.

Strong topology-dependent results are available for homogeneous conjunctive Boolean networks and, by duality, their disjunctive counterparts, where interaction-graph structure and loop number constrain periodic behaviour (Jarrah et al. 2010). These conclusions, however, do not extend automatically to networks in which conjunctive and disjunctive canalizing rules coexist. In particular, mixed rule types can invalidate the homogeneous loop-number divisibility mechanism. At the same time, existing approaches to Boolean-network reduction and attractor analysis—including trap-space methods, feedback-based techniques, model checking, SAT, decision diagrams, and graph-based algorithms—address complementary aspects of the state-space problem (Klarner et al. 2015; Klarner and Siebert 2015; Aracena et al. 2021; Mori and Akutsu 2022; Zheng et al. 2013; Yizengaw 2026). What remains insufficiently developed is a structural periodic-orbit theory that exploits progressively available canalizing information in the mixed conjunctive–disjunctive setting.

This paper addresses that gap for deterministic synchronous Boolean networks whose coordinate rules belong to the specified conjunctive, disjunctive, or mixed nested-canalizing classes. The scope is deliberately restricted: “mixed” refers to coexistence of these structured rule types and does not encompass arbitrary non-monotone Boolean regulation. Complemented canalizing literals can represent certain inhibitory effects, but unrestricted signed or non-monotone logic lies outside the present theory. Likewise, the principal cycle-counting results concern synchronous dynamics; asynchronous dynamics is considered only to identify which reduction properties survive when deterministic evolution is replaced by a transition relation (Aracena et al. 2009; Skodawessely and Klemm 2011; Yizengaw 2026).

The central contribution is a hierarchy of admissibility relations obtained by retaining successively deeper canalizing layers. We prove that the exact reduced dynamics is contained in every level of this hierarchy and that increasing the retained canalizing information can only tighten the resulting periodic-orbit bound, with an exact criterion for strict improvement. On each forward-invariant saturated trap space, the original dynamics is conjugate to a reduced Boolean system, yielding an exact state-space reduction from $2^n$ states to $2^{n-d_T}$ states, where $d_T$ is the number of coordinates frozen by saturation. We further provide a closure algorithm and complexity analysis that distinguish the cost of saturating a specified seed from the potentially combinatorial problem of discovering all relevant trap spaces. Together, these results replace an inappropriate extension of homogeneous loop-number restrictions by a progressively refinable structural bound tailored to mixed canalizing dynamics.

The theoretical results are illustrated by nonconstant disjunctive and genuinely mixed examples and by exhaustive analysis of a synthetic five-node module. To avoid overstating biological generality, traceable curated Boolean models are additionally used as an applicability audit: models falling outside the theorem hypotheses are identified as such rather than treated as empirical validation. This distinction clarifies both the potential and the present limitations of the framework for biological network analysis.

The remainder of the paper is organized as follows. Section “Preliminaries and background” introduces the model class, notation, and background results. Section “Main results” develops the exact reduction, hierarchical cycle bounds, saturation theory, and computational analysis. Section “Applications and biological context” presents the worked examples and biological applicability assessment. Section “Conclusion and future directions” summarizes the main conclusions, limitations, and directions for further work.

## Preliminaries and background

Throughout, let $\mathbb {F}_2=\{0,1\}$ denote the binary field and $[n]:=\{1,\ldots ,n\}$. The state space of an *n*-dimensional Boolean network is $\mathbb {F}_2^n$.

### Definition 2.1

*(Boolean network and synchronous dynamics)* Following the standard finite-state formulation of Boolean networks (Kauffman 1969; Drossel 2008), a Boolean network is a map

$$ F=(f_1,\ldots ,f_n): \mathbb {F}_2^n\longrightarrow \mathbb {F}_2^n, \qquad F(x)_i=f_i(x), $$

where $f_i:\mathbb {F}_2^n\rightarrow \mathbb {F}_2$ is the update rule of coordinate *i*. We consider deterministic synchronous dynamics,

$$ x(t+1)=F(x(t)), \qquad t=0,1,2,\ldots , $$

so all coordinates at time *t+1* are evaluated from the same state *x*(*t*).

### Remark 2.1

*(Update regime)* The main results concern synchronous deterministic dynamics. Asynchronous Boolean dynamics is instead represented by a transition relation and may possess recurrent attractors given by terminal strongly connected components (Aracena et al. 2009; Skodawessely and Klemm 2011; Zheng et al. 2013; Yizengaw 2026). The extension of the present reduction to asynchronous updating is left as a future direction.

### Definition 2.2

*(Periodic orbit and minimal period)* A state $x\in \mathbb {F}_2^n$ is periodic if

$$ F^\ell (x)=x $$

for some integer $\ell \ge 1$ (Drossel 2008). Its minimal period is the least such *ℓ *, and the corresponding periodic orbit is

$$ \mathcal {O}_F(x) = \{x,F(x),\ldots ,F^{\ell -1}(x)\}. $$

We denote by $C(F)_\ell$ the set of distinct periodic orbits of minimal period *ℓ *, counted modulo cyclic rotation, and write

$$ A_\ell (F):=|C(F)_\ell |. $$

A period-one orbit is a fixed point.

### Definition 2.3

*(Dependency digraph and loop number)* The dependency digraph of *F* is $G_F=([n],E)$, where *j→ i* belongs to *E* whenever $f_i$ depends essentially on $x_j$; equivalently, there exist $x,x'\in \mathbb {F}_2^n$ differing only in coordinate *j* such that

$$ f_i(x)\ne f_i(x'). $$

If $G_F$ is strongly connected, its loop number (cyclicity) is

$$ p^*(G_F) := \gcd \{\,|\gamma |: \gamma \text { is a directed cycle of }G_F\,\}. $$

For homogeneous conjunctive and disjunctive Boolean networks, this graph invariant constrains the possible periods (Jarrah et al. 2010).

### Remark 2.2

*(Role of the loop number)* The classical period restrictions associated with $p^*(G_F)$ apply to the homogeneous conjunctive/disjunctive setting (Jarrah et al. 2010). No corresponding universal divisibility condition is assumed for a mixed network; the bounds developed below instead follow from inclusions between exact and progressively refined transition relations.

### Definition 2.4

*(Nested canalizing function)* Following the standard nested-canalizing representation (Jarrah et al. 2007; Li et al. 2013), a Boolean function $f:\mathbb {F}_2^m\rightarrow \mathbb {F}_2$ has canalizing depth *d* if there exist distinct variables $x_{\sigma (1)},\ldots ,x_{\sigma (d)}$, canalizing inputs $a_1,\ldots ,a_d\in \mathbb {F}_2$, canalized outputs $b_1,\ldots ,b_d\in \mathbb {F}_2$, and a residual Boolean rule *ρ * such that

$$ f(x)= {\left\{ \begin{array}{ll} b_1, & x_{\sigma (1)}=a_1,\\ b_2, & x_{\sigma (1)}\ne a_1,\; x_{\sigma (2)}=a_2,\\ \vdots & \\ b_d, & x_{\sigma (s)}\ne a_s\ (s<d),\; x_{\sigma (d)}=a_d,\\ \rho (x), & x_{\sigma (s)}\ne a_s\quad (s=1,\ldots ,d). \end{array}\right. } $$

The tests are evaluated in the displayed order: the *r*th test is reached only if the preceding *r-1* canalizing conditions fail. Consecutive tests having the same canalized output form a *canalizing layer* (Li et al. 2013; Dimitrova et al. 2022).

### Remark 2.3

*(Canalizing representation)* Algorithms for identifying canalizing variables, layers, and related structural decompositions have been developed for standard representations of Boolean functions (Dimitrova et al. 2022). The hierarchical construction below takes the ordered canalizing representation of each local rule as structural input; it does not require such a representation to be recovered as part of the cycle-counting theorem.

### Definition 2.5

*(Conjunctive, disjunctive, and mixed rule types)* For $N_i\subseteq [n]$, the positive conjunctive and disjunctive Boolean rules are, respectively,

$$ f_i(x)=\bigwedge _{j\in N_i}x_j, \qquad f_i(x)=\bigvee _{j\in N_i}x_j. $$

These standard conjunctive and disjunctive rule classes are treated in Jarrah et al. (2010). A network is *conjunctive* or *disjunctive* when all of its coordinate rules are of the corresponding type. In this paper, a *mixed canalizing Boolean network* (MCBN) permits conjunctive and disjunctive nested-canalizing rule types at different coordinates, with any heterogeneous canalizing inputs and outputs specified through the ordered representation of Definition 2.4.

### Remark 2.4

*(Restriction of the model class)* Positive conjunctive and disjunctive rules are monotone with respect to the standard product order on $\mathbb {F}_2^n$. General Boolean models of regulatory systems may contain activating and inhibitory interactions and need not be monotone (Thomas 1973). Complemented canalizing literals can encode certain inhibitory effects, but the present theory does not cover arbitrary non-monotone Boolean logic. All results below are therefore conditional on the stated conjunctive/disjunctive nested-canalizing structure.

### Definition 2.6

*(Trap space)* Let *S⊆ [n]* and let $c=(c_j)_{j\in S}\in \mathbb {F}_2^S$. Define the subcube

$$ T(S,c) := \{x\in \mathbb {F}_2^n: x_j=c_j\text { for every }j\in S\}. $$

It is a *trap space* for *F* if it is forward invariant,

* F(T(S,c))⊆ T(S,c). *

This is the standard forward-invariance notion of a trap space (Klarner et al. 2015; Klarner and Siebert 2015; Yizengaw 2026). If *U=[n]∖ S* denotes the set of free coordinates, then

$$ \dim (T)=|U|, \qquad |T|=2^{|U|}. $$

### Definition 2.7

*(Saturated trap space and saturation codimension)* A trap space obtained when the canalizing closure operator *Φ * introduced in Section Main results reaches a fixed point is called a *saturated trap space*. For such a trap space *T*, define

$$ d_T:=n-\dim (T). $$

We call $d_T$ the *saturation codimension*, equivalently the number of coordinates frozen on *T*. Consequently,

$$ |T|=2^{\,n-d_T}. $$

### Remark 2.5

*(Canalization depth versus saturation codimension)* The intrinsic canalization depth of a local Boolean rule (Li et al. 2013; Dimitrova et al. 2022) and the saturation codimension $d_T$ are distinct quantities. The former describes the ordered canalizing structure of a rule; the latter counts the network coordinates frozen in a particular saturated trap space. No equality between these quantities is assumed.

### Definition 2.8

*(Transition relation and cycle notation)* Let *X* be a finite set and $R\subseteq X\times X$ a directed transition relation. A simple directed cycle of length *ℓ * is a sequence of distinct vertices $x_0,\ldots ,x_{\ell -1}$ satisfying

$$ (x_r,x_{r+1})\in R \quad (0\le r<\ell -1), \qquad (x_{\ell -1},x_0)\in R. $$

Two such sequences represent the same cycle if they differ only by a cyclic rotation. We denote the resulting set of cycles by $C(R)_\ell$.

If *g:X→ X* is deterministic, we identify *g* with its graph

$$ \operatorname {graph}(g) := \{(x,g(x)):x\in X\}. $$

Then $C(g)_\ell$ is precisely the set of periodic orbits of *g* having minimal period *ℓ *.

### Remark 2.6

*(Cycle monotonicity under relation inclusion)* If $R_1\subseteq R_2$, every directed *ℓ *-cycle of $R_1$ is also a directed *ℓ *-cycle of $R_2$. Hence

$$ C(R_1)_\ell \subseteq C(R_2)_\ell , \qquad |C(R_1)_\ell |\le |C(R_2)_\ell |. $$

This elementary inclusion principle is the mechanism underlying the hierarchical cycle bounds in Section Main results.

## Main results

### Definition 3.1

*(Depth-truncated admissibility)* Let $F:\mathbb {F}_2^n\rightarrow \mathbb {F}_2^n$ and let

$$ T=\{x\in \mathbb {F}_2^n:x_j=c_j,\ j\in S\} $$

be a forward-invariant subcube. Put *U=[n]∖ S*, $S_T=\mathbb {F}_2^U$, and let $\iota _T:S_T\rightarrow T$ restore the fixed coordinates. For each *i∈ U*, fix an ordered canalizing representation of $f_i|_T$ of canalizing depth $d_i$, with tests $\lambda _{i,1},\ldots ,\lambda _{i,d_i}$ and corresponding canalized outputs $b_{i,1},\ldots ,b_{i,d_i}$, where $\lambda _{i,r}(x)=1$ means that the *r*th canalizing condition is satisfied (He and Macauley 2016; Li et al. 2013). Set

$$ D_T:=\max _{i\in U}d_i. $$

For $0\le k\le D_T$, define $\Sigma _T^{(k)}\subseteq S_T\times S_T$ by $(y,z)\in \Sigma _T^{(k)}$ if, for every *i∈ U* and $1\le r\le \min \{k,d_i\}$,

$$ \left( \bigwedge _{s<r}\lambda _{i,s}(\iota _T(y))=0\right) \wedge \lambda _{i,r}(\iota _T(y))=1 \quad \Longrightarrow \quad z_i=b_{i,r}. $$

If none of the retained tests is triggered, $z_i$ is unconstrained. Hence

$$ \Sigma _T^{(0)}=S_T\times S_T,\qquad \Sigma _T^{(0)}\supseteq \Sigma _T^{(1)} \supseteq \cdots \supseteq \Sigma _T^{(D_T)}. $$

Thus $\Sigma _T^{(k)}$ retains the first *k* ordered canalizing tests, where available, together with their corresponding outputs.

### Theorem 1

(Exact reduced dynamics) Let *T* be as in Definition 3.1, and define

$$ \pi _T:T\rightarrow S_T,\qquad \pi _T(x)=x_U. $$

Then $\pi _T$ is a bijection with inverse $\iota _T$, and

$$ h_T:=\pi _T\circ F\circ \iota _T:S_T\rightarrow S_T $$

is conjugate to $F|_T$, namely

$$ \pi _T\circ F|_T=h_T\circ \pi _T. $$

Consequently, for every $\ell \ge 1$,

$$ |C(F|_T)_\ell |=|C(h_T)_\ell |, $$

where $C(G)_\ell$ denotes the set of periodic orbits of *G* of minimal period *ℓ *. Moreover,

$$ \operatorname {graph}(h_T)\subseteq \Sigma _T^{(k)}, \qquad 0\le k\le D_T. $$

### Proof

Since the coordinates in *U=[n]∖ S* are free on *T*, $\pi _T$ is bijective with inverse $\iota _T$. Forward invariance, *F(T)⊆ T*, ensures that $h_T$ is well defined. For *x∈ T*,

$$ h_T(\pi _T(x)) =\pi _TF\iota _T\pi _T(x) =\pi _TF(x), $$

and hence

$$ \pi _T\circ F|_T=h_T\circ \pi _T. $$

Thus $\pi _T$ bijectively maps periodic orbits of $F|_T$ onto periodic orbits of $h_T$ while preserving minimal periods, yielding

$$ |C(F|_T)_\ell |=|C(h_T)_\ell |, \qquad \ell \ge 1. $$

Now let $(y,z)\in \operatorname {graph}(h_T)$ and put $x=\iota _T(y)$. Then $z=\pi _TF(x)$, so $z_i=f_i(x)$ for every *i∈ U*. If, for some $r\le \min \{k,d_i\}$,

$$ \lambda _{i,s}(x)=0\quad (s<r), \qquad \lambda _{i,r}(x)=1, $$

then the ordered canalizing representation gives $f_i(x)=b_{i,r}$, and hence $z_i=b_{i,r}$. Thus every defining implication of $\Sigma _T^{(k)}$ is satisfied, proving

$$ \operatorname {graph}(h_T)\subseteq \Sigma _T^{(k)}. $$

*□*

**Relation to exact aggregation and abstraction refinement.** The reduction above also has a broader interpretation beyond Boolean-network-specific reduction techniques. The conjugacy in Theorem 1, and its saturated-trap-space specialization below, can be viewed as a deterministic finite-state instance of exact dynamics-preserving aggregation: after restriction to a forward-invariant subcube, projection onto the free coordinates is bijective and therefore loses no information about the restricted dynamics, including periodic orbits and their minimal periods. This is conceptually related to exact aggregation and lumpability, which provide general frameworks for constructing reduced systems while preserving specified dynamical quantities (Buchholz 1994; Ressi et al. 2024). In particular, Ressi et al. use lumpability-based formal methods to obtain exact behaviour-preserving reductions of structured neural systems (Ressi et al. 2024).

The nested relations

$$ \Sigma _T^{(D_T)}\subseteq \cdots \subseteq \Sigma _T^{(1)}\subseteq \Sigma _T^{(0)} $$

admit a complementary abstraction–refinement interpretation. The coarsest relation deliberately forgets the greatest amount of exact transition information and therefore admits the largest set of behaviours; successive levels restore additional canalizing constraints and remove spurious transitions while continuing to contain $\operatorname {graph}(h_T)$. This parallels the general use of ordered behavioural abstractions and progressively refined transition descriptions. The contribution here is not the general principle of exact aggregation or refinement itself, but its concrete realization from nested-canalizing structure: saturation produces invariant subcubes supporting exact coordinate reduction, while the ordered canalizing layers supply an intrinsic refinement mechanism for the transition over-approximation. We use this comparison at the level of reduction principles rather than identifying the present deterministic conjugacy with any particular stochastic lumpability definition.

### Theorem 2

(Hierarchical canalization bounds) Under the hypotheses of Theorem 1,

$$ \operatorname {graph}(h_T)\subseteq \Sigma _T^{(D_T)} \subseteq \cdots \subseteq \Sigma _T^{(1)} \subseteq \Sigma _T^{(0)}. $$

Hence, for every $\ell \ge 1$,

$$ \boxed { |C(F|_T)_\ell | \le |C(\Sigma _T^{(D_T)})_\ell | \le \cdots \le |C(\Sigma _T^{(0)})_\ell | }. $$

Moreover, for $$0\le k<j\le D_T$$,

$$ |C(\Sigma _T^{(j)})_\ell |<|C(\Sigma _T^{(k)})_\ell | $$

if and only if $\Sigma _T^{(k)}$ contains a directed simple *ℓ *-cycle absent from $\Sigma _T^{(j)}$.

### Proof

Increasing *k* only adds defining constraints, so $\Sigma _T^{(k+1)}\subseteq \Sigma _T^{(k)}$. Together with Theorem 1, this proves the relation chain. Since *R'⊆ R* implies $C(R')_\ell \subseteq C(R)_\ell$, cycle monotonicity and conjugacy give

$$ |C(F|_T)_\ell |=|C(h_T)_\ell | \le |C(\Sigma _T^{(D_T)})_\ell | \le \cdots \le |C(\Sigma _T^{(0)})_\ell |. $$

Finally, for *k<j*, $C(\Sigma _T^{(j)})_\ell \subseteq C(\Sigma _T^{(k)})_\ell$; since $S_T$ is finite, the inequality of cardinalities is strict exactly when

$$ C(\Sigma _T^{(k)})_\ell \setminus C(\Sigma _T^{(j)})_\ell \ne \varnothing , $$

which is precisely the stated cycle-elimination condition. *□ *

### Proposition 3.1

(Exact edge count and quantitative sparsification) For $y\in S_T$ and $0\le k\le D_T$, let

$$\begin{aligned} & I_k(y):= \left\{ i\in U:\exists \,r\le \min \{k,d_i\},\ \lambda _{i,s}(\iota _T(y))\right. \\ & \quad \left. =0\ (s<r),\ \lambda _{i,r}(\iota _T(y))=1\right\} , \qquad m_k(y):=|I_k(y)|. \end{aligned}$$

Then

$$ \deg _{\Sigma _T^{(k)}}^+(y)=2^{|U|-m_k(y)}, \qquad |\Sigma _T^{(k)}| =\sum _{y\in S_T}2^{|U|-m_k(y)}. $$

Moreover, $I_k(y)\subseteq I_{k+1}(y)$ for $k<D_T$, and hence

$$ |\Sigma _T^{(D_T)}|\le \cdots \le |\Sigma _T^{(1)}| \le |\Sigma _T^{(0)}|=2^{2|U|}. $$

Writing $$\Delta _k(y):=m_{k+1}(y)-m_k(y)$$,

$$ |\Sigma _T^{(k)}|-|\Sigma _T^{(k+1)}| = \sum _{y\in S_T} 2^{|U|-m_k(y)} \bigl (1-2^{-\Delta _k(y)}\bigr ). $$

Consequently, for $$0\le k<D_T$$,

$$ |\Sigma _T^{(k+1)}|<|\Sigma _T^{(k)}| $$

if and only if there exist $y\in S_T$ and *i∈ U* with $d_i\ge k+1$ such that

$$ \lambda _{i,s}(\iota _T(y))=0\quad (1\le s\le k), \qquad \lambda _{i,k+1}(\iota _T(y))=1. $$

### Proof

Fix $y\in S_T$. For each $i\in I_k(y)$, the first triggered retained test uniquely fixes $z_i$; for $i\notin I_k(y)$, no retained test is triggered and $z_i$ is free. Thus exactly $|U|-m_k(y)$ target coordinates are free, giving

$$ \deg _{\Sigma _T^{(k)}}^+(y)=2^{|U|-m_k(y)}. $$

Summing over *y* yields the edge-count formula.

Since retaining level *k+1* cannot remove an earlier constraint, $I_k(y)\subseteq I_{k+1}(y)$, proving monotonicity; at *k=0*, $m_0(y)=0$ and $|S_T|=2^{|U|}$, so $|\Sigma _T^{(0)}|=2^{2|U|}$. Furthermore,

$$ 2^{|U|-m_{k+1}(y)} = 2^{|U|-m_k(y)}2^{-\Delta _k(y)}, $$

which gives the stated difference formula. Its summands are nonnegative and one is positive exactly when $m_{k+1}(y)>m_k(y)$. By the ordered canalizing definition, this occurs precisely when some *(k+1)*st test is reached and triggered, i.e.,

$$ d_i\ge k+1,\qquad \lambda _{i,s}(\iota _T(y))=0\ (1\le s\le k),\qquad \lambda _{i,k+1}(\iota _T(y))=1. $$

*□*

### Theorem 3

(Trace bounds for periodic-orbit counts) Let $M_k$ be the adjacency matrix of $\Sigma _T^{(k)}$, indexed by $S_T$. Then, for every $\ell \ge 1$ and $0\le k\le D_T$,

$$ |C(F|_T)_\ell | \le |C(\Sigma _T^{(k)})_\ell | \le \frac{\operatorname {tr}(M_k^\ell )}{\ell }, $$

where for *ℓ =1* the right-hand side is $\operatorname {tr}(M_k)$. Moreover,

$$ \operatorname {tr}(M_{k+1}^\ell ) \le \operatorname {tr}(M_k^\ell ), \qquad 0\le k<D_T,\quad \ell \ge 1. $$

Thus the admissibility hierarchy yields a monotone hierarchy of algebraically computable upper bounds on exact periodic-orbit counts.

### Proof

By Theorem 2, every exact period-*ℓ * orbit induces a simple directed *ℓ *-cycle in $\Sigma _T^{(k)}$, giving the first inequality. Since $\operatorname {tr}(M_k^\ell )$ counts closed directed walks of length *ℓ *, each simple directed *ℓ *-cycle contributes exactly *ℓ * such walks, one from each starting vertex. Hence

$$ \ell \,|C(\Sigma _T^{(k)})_\ell | \le \operatorname {tr}(M_k^\ell ). $$

This also covers *ℓ =1*, for which the trace counts self-loops.

Finally, $\Sigma _T^{(k+1)}\subseteq \Sigma _T^{(k)}$ implies $0\le M_{k+1}\le M_k$ entrywise. For nonnegative matrices,

$$ 0\le M_{k+1}^{\ell }\le M_k^{\ell } $$

entrywise for every $\ell \ge 1$, and taking traces proves the assertion. *□ *

### Theorem 4

(Strong-component period exclusion) Fix $0\le k\le D_T$, and let $Q_1,\ldots ,Q_r$ be the strongly connected components of $\Sigma _T^{(k)}$. Then, for every $\ell \ge 1$,

$$ |C(F|_T)_\ell | \le \sum _{j:\,|Q_j|\ge \ell } |C(\Sigma _T^{(k)}[Q_j])_\ell |. $$

In particular,

$$ \max _{1\le j\le r}|Q_j|<\ell \quad \Longrightarrow \quad C(F|_T)_\ell =\varnothing . $$

Hence fragmentation of the admissibility relation under increasing canalizing depth can certify exclusion of prescribed periods without exact attractor enumeration.

### Proof

Every exact period-*ℓ * orbit induces a simple directed *ℓ *-cycle in $\Sigma _T^{(k)}$. The vertices of a directed cycle lie in a single strongly connected component, which must therefore contain at least *ℓ * vertices. Partitioning the admissible *ℓ *-cycles by their strongly connected components gives the stated bound and, immediately, the exclusion criterion. *□ *

### Remark 3.1

*(Sharpness)* In general,

$$ \operatorname {graph}(h_T)\subsetneq \Sigma _T^{(D_T)}, $$

because $\Sigma _T^{(D_T)}$ leaves $z_i$ unconstrained whenever all canalizing tests of $f_i|_T$ fail and evaluation reaches the residual rule (He and Macauley 2016). Consequently,

$$ |C(h_T)_\ell | = |C(\Sigma _T^{(D_T)})_\ell | $$

if and only if the residual freedom introduces no additional simple directed *ℓ *-cycles. Imposing the residual rules recovers $\operatorname {graph}(h_T)$ exactly.

### Definition 3.2

*(Saturation closure)* For a consistent partial assignment *α *, let

$$\begin{aligned} & {[}\alpha ]:=\{x\in \mathbb {F}_2^n: x_j=\alpha (j),\ j\in \operatorname {dom}(\alpha )\},\\ & \qquad V_i(\alpha ):=\{f_i(x):x\in [\alpha ]\}. \end{aligned}$$

If $V_i(\alpha )=\{b\}$, coordinate *i* is uniformly forced to *b*. Define

$$ P(\alpha ):=\alpha \cup \{(i,b):i\notin \operatorname {dom}(\alpha ),\ V_i(\alpha )=\{b\}\}, $$

and iterate $\alpha ^{(m+1)}=P(\alpha ^{(m)})$. Since the domains increase monotonically in the finite set [*n*], the iteration stabilizes after finitely many steps; write

$$ \alpha ^*=\Phi (\alpha ^{(0)}). $$

If $T=[\alpha ^*]$ satisfies *F(T)⊆ T*, then *T* is called a *saturated trap space*, and

$$ d_T:=|\operatorname {dom}(\alpha ^*)|=n-\dim (T). $$

This closure is a value-percolation construction on Boolean subspaces, while *F(T)⊆ T* is the defining dynamical invariance of a trap space (Klarner et al. 2015; Cifuentes-Fontanals et al. 2025).

### Theorem 5

(Exact reduction after saturation) Let $T=[\alpha ^*]$ be a forward-invariant saturated trap space, and let

$$ d_T=|\operatorname {dom}(\alpha ^*)|. $$

Then

$$ |T|=2^{\,n-d_T}. $$

Moreover, the reduced map

$$ h_T=\pi _T\circ F\circ \iota _T $$

is conjugate to $F|_T$. Consequently, exact periodic-orbit enumeration restricted to *T* may be performed on a state space of size

$$ 2^{\,n-d_T} $$

instead of $2^n$, with all minimal periods and periodic-orbit counts preserved.

### Proof

Since $\alpha ^*$ fixes exactly $d_T$ coordinates, the remaining $n-d_T$ coordinates are free; hence

$$ |T|=2^{\,n-d_T}. $$

Because *T* is forward invariant, Theorem 1 applies and yields a conjugacy

$$ \pi _T\circ F|_T=h_T\circ \pi _T. $$

Thus $\pi _T$ induces a bijection between the periodic orbits of $F|_T$ and those of $h_T$, preserving minimal periods. Therefore exact periodic-orbit enumeration on *T* is equivalent to enumeration on the $2^{\,n-d_T}$ states of $S_T$. *□ *

**Saturation algorithm.** The closure $\Phi (\alpha ^{(0)})$ can be computed by value percolation (Cifuentes-Fontanals et al. 2022, 2025). For a local rule represented by an ordered canalizing decision list, $V_i(\alpha )$ is obtained recursively from the residual branch outward: a fixed triggering literal selects its canalized output, a fixed non-triggering literal passes to the suffix, and an unfixed literal takes the union of the triggered and suffix outputs. Thus, if the residual output set is available in *O*(1) time, one scan costs $O(q_i)$, where $q_i$ is the stored canalizing depth; otherwise the cost of evaluating the residual rule must be added (He and Macauley 2016).

**Algorithm 1:**
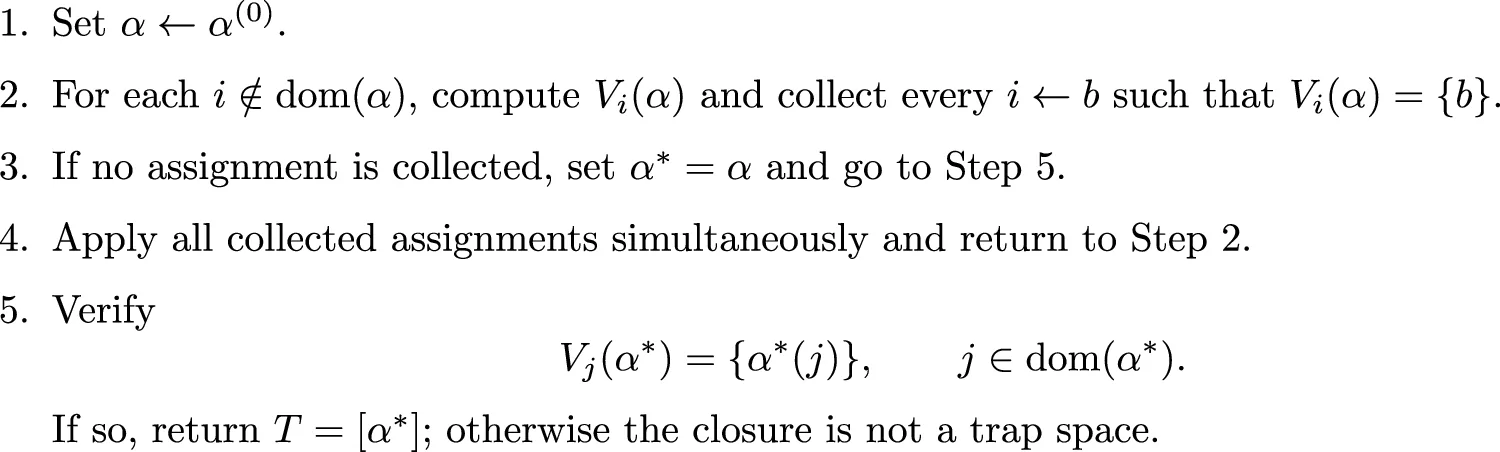
Saturation closure

### Proposition 3.2

(Saturation cost) Assume that each stored canalizing decision list *i* can be scanned in $O(q_i)$ time, and set

$$ \Lambda :=\sum _{i=1}^n q_i. $$

Then a direct implementation of Algorithm 1 computes the saturation closure of a specified seed in

* O(nΛ ) *

time and *O(n+Λ )* memory.

### Proof

One full pass over all local rules costs

$$ O\!\left( \sum _{i=1}^n q_i\right) =O(\Lambda ). $$

Each nonterminal pass fixes at least one previously free coordinate; hence there are at most $n-|\operatorname {dom}(\alpha ^{(0)})|\le n$ such passes, followed by at most one terminal verification pass. Therefore the total running time is *O(nΛ )*. The decision lists require *O(Λ )* memory, while the partial assignment, collected updates, and auxiliary bookkeeping require *O*(*n*) memory, yielding *O(n+Λ )* overall. *□ *

### Remark 3.2

*(Scope of the computational reduction)* Proposition 3.2 concerns the closure of a specified seed and does not imply polynomial-time enumeration of all trap spaces. Since each coordinate may be fixed to 0, fixed to 1, or left free, the space of partial assignments has cardinality

$$ 3^n $$

in the worst case. Hence Theorem 5 yields a computationally effective reduction when relevant trap spaces can be identified without exhaustive seed enumeration and when $d_T$ is large enough that the reduced state space

$$ 2^{\,n-d_T} $$

is computationally tractable.

## Applications and biological context

This section illustrates two consequences of the theory: the progressive tightening of periodic-orbit bounds under retained canalizing information and the exact state-space reduction induced by saturation. The synthetic examples serve only to verify the mathematical claims; biological interpretation is restricted to curated models satisfying the rule and update assumptions of Sect. “Preliminaries and background”.

**Disjunctive dynamics.** Consider

$$ x_1'=x_3\vee x_4,\qquad x_2'=x_3\vee x_4,\qquad x_3'=x_1\vee x_2,\qquad x_4'=x_1\vee x_2 . $$

Every displayed input is essential, and

$$ (1,1,0,0)\longmapsto (0,0,1,1) \longmapsto (1,1,0,0), $$

so the system has a periodic orbit of minimal period 2. Its dependency digraph is strongly connected with loop number 2, consistent with the classical homogeneous disjunctive theory (Jarrah et al. 2010). Thus nonconstant disjunctive canalization is compatible with nontrivial recurrent dynamics.

**Mixed dynamics and failure of homogeneous period restrictions.** Consider

$$ x_1'=x_2\wedge x_3,\qquad x_2'=x_1\wedge x_3,\qquad x_3'=x_1\vee x_2. $$

The first two rules are conjunctive, the third is disjunctive, and all displayed inputs are essential. Moreover,

$$ (0,1,1)\longmapsto (1,0,1) \longmapsto (0,1,1). $$

The dependency digraph contains directed cycles of lengths 2 and 3, so its cycle-length gcd is 1. Nevertheless, the synchronous dynamics has a period-2 orbit. Hence the homogeneous loop-number divisibility restriction does not extend to this mixed class, motivating the relation-based bounds of Theorem 2.

**Exhaustive verification on a mixed network.** Consider

$$ \begin{aligned} A'&=C\vee E,&B'&=C\vee D,\\ C'&=A\wedge B,&D'&=A\wedge C,\\ E'&=B\wedge C . \end{aligned} $$

This synthetic system is used solely as an exactly enumerable mixed instance. Exhaustive evaluation of its $2^5=32$ states yields the fixed points

$$ \textbf{00000},\qquad \textbf{11111}, $$

the period-2 orbit

$$ \{\textbf{00100},\textbf{11000}\}, $$

and the period-4 orbit

$$ \{\textbf{01100},\textbf{11001}, \textbf{10100},\textbf{11010}\}. $$

Hence

$$ (A_1,A_2,A_3,A_4)=(2,1,0,1). $$

Since $\Sigma ^{(0)}=S\times S$ and *|S|=32*, it is the complete directed relation on 32 states. Therefore

$$ |C(\Sigma ^{(0)})_1|=32, \qquad |C(\Sigma ^{(0)})_\ell | =\frac{(32)_\ell }{\ell }, \quad \ell \ge 2, $$

and hence

$$\begin{aligned} & \left( |C(\Sigma ^{(0)})_1|, |C(\Sigma ^{(0)})_2|, |C(\Sigma ^{(0)})_3|, |C(\Sigma ^{(0)})_4| \right) \\ & \quad = (32,496,9920,215760). \end{aligned}$$

Thus

$$ A_\ell \le |C(\Sigma ^{(0)})_\ell |, \qquad \ell =1,\ldots ,4, $$

with substantial slack, as expected for the maximally permissive relation.

The quantity relevant to Theorem 2 is the full hierarchy

$$ A_\ell \le |C(\Sigma ^{(D_T)})_\ell | \le \cdots \le |C(\Sigma ^{(1)})_\ell | \le |C(\Sigma ^{(0)})_\ell |. $$

For this example every local rule has canalizing depth two, so $D_T=2$ on the full state space. Using the natural ordered canalizing representations of the two-input OR and AND rules and exhaustively enumerating simple directed cycles in the corresponding admissibility relations gives

$$ \begin{aligned}&\bigl (|C(\Sigma ^{(1)})_1|,|C(\Sigma ^{(1)})_2|, |C(\Sigma ^{(1)})_3|,|C(\Sigma ^{(1)})_4|\bigr )\\&=(13,78,453,3145),\\&\bigl (|C(\Sigma ^{(2)})_1|,|C(\Sigma ^{(2)})_2|, |C(\Sigma ^{(2)})_3|,|C(\Sigma ^{(2)})_4|\bigr )\\&=(8,19,33,58). \end{aligned} $$

Consequently, for each $\ell =1,\ldots ,4$ the hierarchy is strictly tightened at both retained depths:

$$ A_\ell<|C(\Sigma ^{(2)})_\ell |<|C(\Sigma ^{(1)})_\ell | <|C(\Sigma ^{(0)})_\ell |. $$

Thus the example directly verifies the monotone improvement predicted by Theorem 2: retaining the first canalizing test removes a large fraction of the spurious cycles of the free relation, and retaining the second test tightens the bound further. The deepest relation is still not cycle-exact, consistently with Remark 3.1 and the residual-rule freedom described there. Table 1 summarises the exactperiodic-orbit counts and the certified upper bounds at all three admissibility levels for this five-node mixed network.

**Table 1:** Exact periodic-orbit counts and the complete hierarchy of certified admissibility-relation bounds for the five-node synthetic MCBN

Period *ℓ *	1	2	3	4
Exact $A_\ell$	2	1	0	1
$|C(\Sigma ^{(2)})_\ell |$	8	19	33	58
$|C(\Sigma ^{(1)})_\ell |$	13	78	453	3145
$|C(\Sigma ^{(0)})_\ell |$	32	496	9920	215760

**Exact reduction by saturation.** Let *T* be a forward-invariant saturated trap space with saturation codimension $d_T$. By Theorem 5, the exact dynamics on *T* is conjugate to a Boolean system on

$$ 2^{\,n-d_T} $$

states. Relative to the unreduced state space, the reduction factor is therefore

$$ \frac{2^n}{2^{\,n-d_T}}=2^{d_T}. $$

This identity gives the appropriate quantitative measure of the reduction: each additional coordinate certified as frozen divides the remaining exact state space by two.

The statement is conditional on a specified saturated trap space and does not imply efficient enumeration of all minimal trap spaces. Consequently, computational validation of the reduction should report, for every specified seed or input condition,

$$ \bigl ( n,\ |E|,\ d_T,\ 2^n,\ 2^{n-d_T},\ t_{\textrm{sat}},\ t_{\textrm{attr}},\ \{A_\ell \}_{\ell \ge 1} \bigr ), $$

where $t_{\textrm{sat}}$ is the saturation time and $t_{\textrm{attr}}$ is the time required for exact attractor analysis on the reduced state space. Values of $d_T$ and runtime are reported only when they have been obtained from an explicit computation.

**Biological applicability.** The structural premise of the theory has empirical motivation. Large-scale analysis of expert-curated Boolean gene-regulatory models shows that nested canalization is highly prevalent (Kadelka et al. 2024b), while related work links canalization to reduced regulatory nonlinearity (Kadelka et al. 2024a). This prevalence supports studying canalizing structure, but it does not imply that every curated network belongs to the MCBN class considered here.

A curated model is therefore treated as theorem-eligible only if: (i) the relevant local rules admit the conjunctive/disjunctive nested-canalizing representation of Definitions 2.4–2.5; (ii) the dynamics under comparison is deterministic and synchronous; and (iii) any subspace used for exact reduction is verified to be forward invariant. Arbitrary signed non-monotone rules, probabilistic updates, and asynchronous semantics are not silently replaced by MCBN dynamics.

This distinction excludes several biologically important models from direct theorem-level benchmarking in their published form. The mammalian cell-cycle model of Fauré et al. (2006), the CD4$^+$ T-cell differentiation model of Puniya et al. (2018), and the T-LGL survival-signalling model of Zhang et al. and its subsequent reduction (Zhang et al. 2008; Saadatpour et al. 2011) contain rule or update features that fall outside at least one of the hypotheses used here. They therefore serve as scope tests, not as numerical validations of Theorems 2 and 5.

The biological claim of the present work is consequently structural rather than universal: whenever a curated synchronous Boolean model, or a rigorously justified invariant restriction of one, satisfies the MCBN hypotheses, the hierarchical cycle bounds and saturation reduction apply without alteration. Determining how large this theorem-eligible subclass is within existing model repositories is an empirical question and is not inferred from the prevalence of canalization alone.

## Conclusion and future directions

This work develops a structural framework for bounding synchronous periodic dynamics in Boolean networks that combine conjunctive and disjunctive nested-canalizing rules. The principal result is a hierarchy of transition admissibility relations obtained by retaining successively deeper canalizing information. These relations contain the exact reduced dynamics and yield monotonically tightening certified upper bounds on periodic-orbit counts, with strict improvement characterized by the elimination of admissible cycles. The framework therefore replaces homogeneous loop-number restrictions, which do not extend universally to mixed networks, by bounds that explicitly exploit the ordered canalizing structure of the local rules.

A complementary result concerns exact reduction on forward-invariant saturated trap spaces. If saturation fixes $d_T$ coordinates, the restricted dynamics is conjugate to a Boolean system on $2^{n-d_T}$ states, preserving periodic orbits and their minimal periods. The resulting reduction can be exponential in $d_T$, although this does not imply efficient enumeration of all relevant trap spaces. Together, the hierarchical bounds and saturation reduction separate two distinct benefits of canalizing structure: progressively sharper control of admissible periodic dynamics and exact compression of the state space on invariant subcubes.

The scope of these conclusions is deliberately restricted. The cycle-counting theory assumes deterministic synchronous updating and the specified mixed nested-canalizing rule class; it does not cover arbitrary non-monotone Boolean regulation. The synthetic examples verify the constructions within this class, whereas the curated-model audit identifies biological models for which the hypotheses cannot be assumed. Consequently, biological scalability is not claimed without rule-level eligibility and explicit computation of the corresponding saturation and attractor statistics.

Several extensions follow naturally from these limitations. A first theoretical problem is to characterize structural conditions under which the deepest admissibility relation is cycle-exact, thereby sharpening the role of residual Boolean rules in determining equality in the hierarchical bounds. A second is to develop an asynchronous analogue in which periodic orbits are replaced by recurrent classes of the asynchronous transition graph and to determine which parts of the present admissibility hierarchy remain valid. On the computational side, the principal empirical task is to benchmark theorem-eligible curated networks of increasing size, reporting the attained saturation codimension, state-space reduction, hierarchical bound tightening, runtime, and attractor structure. Extending the framework beyond the present monotone or structured-canalizing setting to signed and genuinely non-monotone regulation constitutes a broader theoretical challenge.

## Data Availability

No datasets were generated or analysed during the current study.
